# A review and content analysis of engagement, functionality, aesthetics, information quality, and change techniques in the most popular commercial apps for weight management

**DOI:** 10.1186/s12966-016-0359-9

**Published:** 2016-03-10

**Authors:** Marco Bardus, Samantha B. van Beurden, Jane R. Smith, Charles Abraham

**Affiliations:** Department of Health Promotion and Community Health, American University of Beirut, Riad El Solh, Beirut, 1107 2020 Lebanon; Psychology Applied to Health group, University of Exeter Medical School, St Luke’s Campus, Heavitree Road, Exeter, EX1 2LU United Kingdom

**Keywords:** Smartphone, Mobile apps, Mobile health (mhealth), Behaviour change techniques, Weight loss, Weight management

## Abstract

**Background:**

There are thousands of apps promoting dietary improvement, increased physical activity (PA) and weight management. Despite a growing number of reviews in this area, popular apps have not been comprehensively analysed in terms of features related to engagement, functionality, aesthetics, information quality, and content, including the types of change techniques employed.

**Methods:**

The databases containing information about all Health and Fitness apps on GP and iTunes (7,954 and 25,491 apps) were downloaded in April 2015. Database filters were applied to select the most popular apps available in both stores. Two researchers screened the descriptions selecting only weight management apps. Features, app quality and content were independently assessed using the Mobile App Rating Scale (MARS) and previously-defined categories of techniques relevant to behaviour change. Inter-coder reliabilities were calculated, and correlations between features explored.

**Results:**

Of the 23 popular apps included in the review 16 were free (70 %), 15 (65 %) addressed weight control, diet and PA combined; 19 (83 %) allowed behavioural tracking. On 5-point MARS scales, apps were of average quality (Md = 3.2, IQR = 1.4); “functionality” (Md = 4.0, IQR = 1.1) was the highest and “information quality” (Md = 2.0, IQR = 1.1) was the lowest domain. On average, 10 techniques were identified per app (range: 1–17) and of the 34 categories applied, goal setting and self-monitoring techniques were most frequently identified. App quality was positively correlated with number of techniques included (*rho* = .58, *p* < .01) and number of “technical” features (*rho* = .48, *p* < .05), which was also associated with the number of techniques included (*rho* = .61, *p* < .01). Apps that provided tracking used significantly more techniques than those that did not. Apps with automated tracking scored significantly higher in engagement, aesthetics, and overall MARS scores. Those that used change techniques previously associated with effectiveness (i.e., goal setting, self-monitoring and feedback) also had better “information quality”.

**Conclusions:**

Popular apps assessed have overall moderate quality and include behavioural tracking features and a range of change techniques associated with behaviour change. These apps may influence behaviour, although more attention to information quality and evidence-based content are warranted to improve their quality.

**Electronic supplementary material:**

The online version of this article (doi:10.1186/s12966-016-0359-9) contains supplementary material, which is available to authorized users.

## Background

There are thousands of apps available in the health and fitness categories of iTunes and Google Play (GP). The most popular apps are used for tracking physical activity (PA) (38 %), diet (31 %), and managing weight (12 %). With so many apps available, it has become challenging to identify those with most potential to support weight loss and reduce obesity [1, 2]. While users select health apps according to perceived design quality and ease of use [3], researchers have so far evaluated apps in terms of more scientific parameters, including adherence to evidence-base and theoretical principles in addition to formal evaluations of design quality. In our recent systematic scoping review of literature on mobile phone and Web 2.0 technologies for weight management [4], we identified 20 reviews and content analyses of apps [5–24]. The majority of these (14/20, 70 %) assessed the presence of theoretical components [5–9, 11, 13, 14, 16, 18, 21–23]; the remainder focused either on evaluating the apps’ design and usability qualities [12, 19, 20, 24], or technical functionalities (e.g., behavioural tracking) [10, 17]. The reviews of theoretical components assessed in particular the presence of constructs derived from behavioural theories [6, 9, 22, 23], tailoring principles [5], evidence-based strategies [7, 16, 18, 21], or techniques designed to promote behaviour change [8, 11, 13, 14], without evaluating functionality and usability. Conversely, the reviews that investigated features related to usability or functionality did not consider theoretical components. Hence, a comprehensive evaluation of the quality of such apps and whether there are relationships between these aspects is lacking.

With regards to behavior change potential, the review evidence specifically analysing behavioural content in weight management apps is limited. The majority of available reviews focus on PA and fitness apps, so it is unclear whether findings from these might extend to broader weight loss apps that do not just focus on PA. Recent reviews demonstrated the importance of incorporating diet in behavioural interventions for weight management, as PA alone has shown only modest effects [25], whereas dietary restriction is an effective weight loss strategy [26]. Additionally, diet combined with PA has beneficial effects on weight maintenance and prevention of weight regain [25–27], which is also aligned with current public health recommendations (e.g., NICE guidelines) for weight management [28]. Indeed, only one review analysed the presence of behavioural components by including both PA and dietary apps [11], but included only apps available on iTunes until November 2012. Even though iTunes App Store covers a large and important part of the apps market, Google Play store recently overtook iTunes by offering a number of apps larger than iTunes [29, 30]. Also, recent industry reports show that Android’s market share accounted for more than 81 % in 2014, compared to the 15 % obtained by Apple iOS [31, 32].

A fruitful stream of research has developed a theory-based classification system (taxonomy) that identified and classified various change techniques (also referred as “behaviour change techniques”), designed to influence a variety of psychological processes and mechanisms underpinning behaviour change [33, 34]. Several systematic reviews of weight loss interventions in general have identified various techniques, which were associated with greater effectiveness [35]. These include goal setting, feedback, and self-monitoring [2, 36, 37]. A recent meta-analysis also found larger effects for weight loss interventions incorporating modelling techniques categorised as “demonstration of behaviour” [37]. The four reviews that explicitly assessed technique content in PA and fitness apps [8, 11, 13, 14] identified varying numbers of techniques, ranging from 1 [8] to 18 per app [11]. The most frequently identified technique types were: prompting goal setting, facilitating self-monitoring, and provision of feedback [11, 13, 14], as well as “demonstration of behaviour”, and “prompting or facilitating social support seeking” [8, 11]. Inclusion of defined technique types has only been assessed in PA and fitness apps so it remains unclear whether weight management apps, more generally, employ the same technique types, or whether these are only used in PA apps.

Additionally, the reviews assessing “usability” of apps provide evaluations based on heuristics for general user interfaces developed by Jacob Nielsen almost 20 years ago [38, 39], or use inductively-developed approaches [12, 17]. They provide generally descriptive, qualitative evaluations of users’ ratings, comments or feedback, and none utilise instruments specifically developed to assess the design and usability of smartphone apps, such as the Mobile App Rating Scale (MARS) [40], which provides app quality ratings for engagement, functionality, aesthetics, and information quality. This instrument has been validated with mental health apps [40] but has not been applied to weight management apps.

To address these gaps, the aim of this study was to evaluate both the quality and content of popular weight management apps available from both iTunes and GP, to answer the following research questions: (1) What is the overall quality of these apps in terms of engagement, functionality, aesthetics, and information quality? (2) What type of change techniques are included in these apps? (3) What are the relationships between user ratings, app quality, other app features, and techniques included, specifically techniques previously found to be associated with weight loss?

## Methods

### Sources of information, selection and coding procedure

App selection proceeded in several steps. First, the mHealthApps repository [41], available in the first quarter of 2015 was downloaded and imported into Excel. The repository included details about all apps available in the Health and Fitness category for both iTunes and GP (US stores). Second, a set of database filters was used to include only popular apps, based on the number of downloads and average user ratings. Although app stores do not provide detailed download information, it is possible to estimate this using formulas proposed by Garg and Telang [42], as a function of an app’s ranking. On GP, apps were ranked according to average user ratings, weighted by the number of ratings and the 15-level ordinal category ‘number of installs’ (ranging from 1 = ‘1–5’ to 15 = ‘10,000,000–50,000,000’). On iTunes, apps were ranked according to average rating weighted by the number of reviews, as the repository does not include information about number of installs.

Third, apps were excluded if they: (a) had an estimated number of downloads below 100, (b) received user ratings below 4 (as done elsewhere [6, 7, 9]), (c) were not available on both iTunes and GP and (d) were free apps with limited functionality which is only unlocked by purchasing the full version (i.e., “freemium”) [22]. In GP, freemium apps were filtered out as the database contains the variable “in-app purchases”; in iTunes in-app purchases were manually checked.

**Fig. 1:**
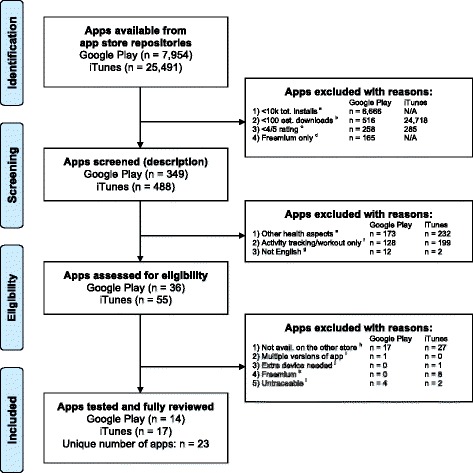
Flow chart of the selection process for apps included in the review. Legend: ^a^ Apps that were downloaded less than 10,000 times. In Google Play, the category ‘Installs’ includes the information 15 levels ranging from “1–5” to “10,000,000–50,000,000”. A popularity index, based on the category of installs, was determined to estimate the number of downloads, as described in Garg and Telang’s formula [53]. ^b^ Apps that were downloaded less than 100 times a day, based on the rank of the apps. ^c^ Apps that received a rating below 4. ^d^ Apps that were classified as having “in-app purchases” (i.e., " freemium"). ^e^ Apps that addressed other health aspects different from weight management or related behaviours (diet and PA), such as smoking, mental health, pregnancy, etc. ^f^ Apps that were workout or activity tracking apps without the aim to weight loss. ^g^ Apps whose description was not in English. ^h^ Apps that did not have a respective counterpart on the other app store. ^i^ Apps that had more than one version (e.g., HD, lite, pro); the basic, fully-functional version was chosen. ^j^ Apps that required an external device (e.g., monitor, wrist band) to function. ^k^ Apps that were either free or paid but the paid version did not have additional and fully functional features. ^l^ Apps that were not available to download after the selection or that were not available for download on the respective devices iPhone 5S (iOS 9.0.2) and Samsung Galaxy S4, GT-I9505 (Android 5.0.2)

Fourth, the first author and a collaborator read the descriptions of the apps and applied further inclusion criteria. Apps were included if they addressed “weight management”, which consists of both PA and dietary behavioural strategies [28], considering the limited role of PA and the predominant role of dietary strategies for effective weight loss [25, 27], and the importance of the combination of PA and diet for long-term effects on weight [28]. This allowed the exclusion of apps that focused only on PA and fitness. We also excluded apps that focused on other aspects of health (maternal health, mental health, etc.). Apps were excluded if their description was not available in English. The selection process is summarised in Fig. 1.

Lastly, two reviewers downloaded the selected apps and independently tested them using an iPhone 5S (iOS 9.0.2) and a Samsung Galaxy S4, GT-I9505 (Android 5.0.2). They first familiarised themselves with the app, used it for approximately 2 days, and independently documented features and evaluated the apps using online forms.

### Data extraction, evaluation criteria and instruments

General and technical information were extracted for descriptive purposes. App name, identification number (ID), version, producer, price, average ratings, and total ratings were retained from the databases. General aspects included “behavioural focus” (i.e., weight management, diet or PA). Technical features included the ability to: track behaviour manually or semi-automatically, share on social media; have an app community; protect data with password; limit access by requiring login; have app reminders; function without web access; work in the background. The total number of features was calculated (range: 0–7), as used in the Mobile App Rating Scale study [40].

App quality was evaluated using the MARS scale [40], which includes 19 items grouped in four domains: 1) engagement (entertainment, interest, customisation, interactivity, and target group); 2) functionality (performance, ease of use, navigation, gestural design); 3) aesthetics (layout, graphics, visual appeal); 4) information quality (accuracy of app description, goals, quality and quantity of information, visual information, credibility, evidence base). All items are measured on a 5-point scale (1 = inadequate to 5 = excellent). A score for each domain is computed as the mean of the items in that domain; an overall score is computed as an average across the domains [25].

The presence or absence of techniques used to promote change was assessed. Categories of change techniques were derived from systematic reviews of weight loss interventions [2, 36, 37] and similar recent reviews of apps for diet and PA [8, 11, 13, 14]. Definitions were derived from taxonomies presenting definitions of technique types [33, 34] and 34 relevant categories were applied. Definitions were adopted form one taxonomy [32] and were modified, where necessary, to allow meaningful technique categorisation. Relevant categories were retained to ensure comprehensive coding of app content and the number of included techniques per app was calculated (Additional file 1: Table S1). Binary variables indicating the presence or absence of technique types found to be associated with effectiveness (i.e., goal setting, self-monitoring, feedback [33, 37, 43]) were included in the analyses.

### Interrater reliabilities

Gwet’s AC1 was used to assess agreement in the selection process [44]. Ordinal and nominal Krippendorf’s alphas (*Kalpha*) [45] were used to estimate inter-coder reliability for the MARS scales and change technique categorisation. Reliability estimates below .70 were discussed to check whether differences existed between operative systems or in the actual application of the scales. Disagreements were solved through discussion. Reliability estimates and correlations are provided in Additional file 1: Tables S1 and Additional file 2: Table S2.

### Analyses

Spearman’s correlations were used to explore the relationships among popularity/ratings, total features, MARS scores, and number of techniques. Independent samples t-tests and one-way ANOVAs (or non-parametric alternatives where indicated) were used to test differences among identified app categories. All tests were carried out in *R* (v. 3.2.2).

## Results

### Database filtering and screening results

The GP and iTunes repositories contained 7,954 and 25,491 apps, respectively. Database filtering left 349 and 488 apps for screening, with 313 apps subsequently excluded from GP (99 % agreement, AC1 = .98, 95 % CI = .97–1.00) and 433 apps from iTunes (99 % agreement, AC1 = .99, 95 % CI = .98–1.00). The selection process and reasons for exclusion are summarised in Fig. 1. The final sample consisted of 23 unique apps.

### General characteristics of the selected apps

Sample descriptive data are summarised in Table 1, while app characteristics are presented in Additional file 3: Table S3. Most of the apps were free (16/23, 70 %). The average price of paid apps was $3.49 on iTunes and $2.99 on GP. The median user rating was 4.5 (IQR = .5) on iTunes and 4.2 (IQR = .6) on GP. The best-rated apps on iTunes were *YouFood* and *Weilos*, while *Ultimate Food Value Diary* was the best on GP. The median number of ratings was 1,436.5 (IQR = 5,186) on iTunes and 3,784 (IQR = 9037) on GP. *MyFitnessPal* received the most ratings on both stores. No differences were detected between paid and free apps with regards to user ratings, number of ratings, app quality, number of techniques, and number of features.

**Table 1:** Descriptive data for the reviewed apps

	n (%)	M (SD)	Md (IQR)	Range
App basic descriptive information				
Paid apps on iTunes, and price ($)	6 (26 %)	3.49 (.55)	3.49 (1.0)	2.99–3.99
Paid apps on GP, and price ($)	5 (22 %)	3.27 (.70)	2.99 (1.0)	2.99–3.99
Avg. user rating on iTunes (scale: 1–5)	22 (96 %)	4.3 (.6)	4.5 (.5)	2.0–5.0
Avg. user rating on GP (scale: 1–5)	23 (100 %)	4.1 (.6)	4.2 (.6)	2.4–4.7
Number of ratings on iTunes (count)	22 (96 %)	23380.7 (92819.4)	1436.5 (5186)	11–438279
Number of ratings on GP (count)	23 (100 %)	48257.0 (174679.8)	3784 (9037)	31–836597
Number of technical features per app (0–7)	23 (100 %)	4.4 (2.2)	5 (4)	1–12
Presence of technical features				
Allows behavioural tracking	19 (83 %)	-	-	-
Manual and semi-automated tracking	9 (82 %)	-	-	-
Allows sharing	8 (9 %)	-	-	-
Has app community	14 (15 %)	-	-	-
Requires login	13 (14 %)	-	-	-
Password	10 (11 %)	-	-	-
Works in background	14 (15 %)	-	-	-
Notifications	18 (20 %)	-	-	-
Needs internet to work	15 (16 %)	-	-	-
MARS app quality ratings (1–5)				
Engagement	23 (100 %)	3.0 (.9)	2.8 (1.2)	1.3–5.0
Functionality	23 (100 %)	3.8 (.9)	4.0 (1.1)	1.8–5.0
Aesthetics	23 (100 %)	3.4 (1.2)	3.8 (2.7)	1.5–4.8
Information quality	23 (100 %)	2.2 (.7)	2.0 (1.1)	1.2–4.1
Total score	23 (100 %)	3.1 (.8)	3.2 (1.4)	1.9–4.6
Number of change techniques	23 (100 %)	9.3 (4.0)	10.0 (6.0)	1.0–17.0
Presence of effective techniques				
Allows goal setting (GS) only	1 (4 %)	-	-	-
GS and self-monitoring (SM)	2 (9 %)	-	-	-
GS, SM and feedback (F)	16 (70 %)	-	-	-
GS, SM, F and description of behaviour (DB)	2 (9 %)	-	-	-
SM only	1 (4 %)	-	-	-
SM and F	1 (4 %)	-	-	-

Notes: *GP* google play, *MARS* mobile app rating scale, *GS* goal setting, *SM* self-monitoring, *F* feedback, *DB* description of behaviour

### Features of the selected apps

The Android and iOS versions of the apps presented quite different technical features, as shown by lower reliability estimates (Md *Kalpha* = .50, IQR = .55). Only the features that were identified by both reviewers were counted. The median number of features offered by the apps was 5 out 7, ranging from 0 (*Fast Food Nutrition & Weight Loss*) to 7 (*NexTrack*). Most apps offered notifications (18/23, 82 %), needed web access to function (15 apps, 68 %), worked in background, and had a community (14 apps, 64 %).

Nineteen apps (83 %) allowed “behavioural tracking”; the remaining four were food information apps (*Fast Food Nutrition & Weight Loss* and *Foods That Burn Fat*), and weight-loss communities (*Weilos* and *YouFood*). Among behavioural tracking apps, 13 (68 %) allowed tracking of weight, diet, and PA, while the remaining six allowed tracking of only one or two of these. Behavioural tracking was either manual (10 apps, 53 %) or semi-automated (9 apps, 47 %). Examples of semi-automated logging included: syncing weight information from digital scales such as Withings (e.g., *Lark, MyDietDiary*); gathering activity information through built-in motion sensors and GPS (e.g., *Lark*, *MyFitnessPal),* or third-party devices such as FitBit; food information through barcode scanners (e.g., *MyPlate*, *CalorieCount*), speech recognition (*Calorie Counter*), or natural language processing (*Lark*).

### App quality

Reliability estimates for quality were good (Md *Kalpha* = .80, IQR = .14). The average MARS score was 3.1 out of 5 (IQR = 1.4), ranging from 1.9 (*Diet Plan* and *Diet Watchers Diary*) to 4.9 (*My Diet Coach PRO*). “Functionality” was the highest-scoring domain (Md = 4.0, IQR = 1.1), followed by “aesthetics” (Md = 3.8, IQR = 2.7), “engagement” (Md = 2.8, IQR = 1.2), and “information quality” (Md = 2.0, IQR = 1.1). All domains were significantly and positively associated with one another, except for information quality with functionality (see Table 2).

**Table 2:** Correlations among app ratings, MARS, number of techniques and total number of features

Variables	1	2	3	4	5	6	7	8	9	10
1. Avg. rating iTunes	1.00									
2. Avg. rating GP	.16	1.00								
3. User ratings iTunes	.27	-.05	1.00							
4. User ratings GP	-.02	.40	.22	1.00						
5. MARS engagement	.47*	.24	.33	.25	1.00					
6. MARS functionality	-.01	-.01	.08	.25	.62**	1.00				
7. MARS aesthetics	.31	.26	.21	.29	.81**	.80**	1.00			
8. MARS information quality	-.12	.29	-.06	.22	.28	.54**	.50*	1.00		
9. MARS total score	.24	.18	.21	.26	.82**	.90**	.96**	.58**	1.00	
10. Number of change techiques	.06	.00	.51*	.18	.49*	.48*	.55**	.47*	.58**	1.00
11. Number of features	.19	.07	.63**	.51*	.64**	.33	.52*	.07	.48*	.61**

Notes: *GP* google play, *MARS* mobile app rating scale. ** *p* < .01; * *p* < .05

### Change technique categories

Reliability estimates for technique coding were good (Md *Kalpha* = .84, IQR = .20). The average number of techniques per app was 10 (IQR = 6), ranging from 1 (*Fast Food Nutrition & Weight Loss*) to 17 (*My Diet Coach PRO*). At least one instance was identified for 24 of the 33 technique categories applied. The most frequently identified technique types were: “self-monitoring” (of behaviour: 20 apps, 87 %; of outcome: 19 apps, 83 %), “goal setting” (of outcome: 19 apps, 83 %; of behaviour: 13 apps, 57 %), and "feedback" (of outcomes: 17 apps, 74 %; of behaviour 16 apps, 70 %). *Lark* and *My Diet Coach PRO*, which were based on interactive coaching, included a higher number of techniques than other apps. Technique categories and frequency of identified instances are presented in Additional file 1: Table S1.

### Relationship between app quality, features and technique inclusion

Number of included techniques was positively associated with overall MARS score (*rho* = .57, *p* < .01), engagement (*rho* = .49, *p* < .05), functionality (*rho* = .48, *p* < .05), aesthetics (*rho* = .55, *p* < .01), and number of ratings on iTunes (*rho* = .51, *p* < .05). Number of features was associated with number of ratings on GP (*rho* = .51, *p* < .05) and on iTunes (*rho* = .63, *p* < .01), MARS score (*rho* = .48, *p* < .05), engagement (*rho* = .64, *p* < .01), aesthetics (*rho* = .52, *p* < .05), and number of techniques (*rho* = .61, *p* < .01). Other correlations were non-significant (see Table 2).

Some general and technical features were related to significant differences in MARS scores and number of techniques. Apps that provided semi-automated tracking included a significantly higher number of techniques (t = 2.93, df = 21, *p* < .01, 95 % CI = 1.59 to 9.43), and had significantly higher MARS scores (t = 2.20, df = 17, *p* = .04, 95 % CI = 1.49 to .03), engagement (t = 2.14, df = 17, *p* = .05, 95 % CI = 1.55 to .01), and aesthetics (U = 16.5, z = −2.34, *p* = .02). Apps that allowed sharing on social media scored higher on overall MARS score (t = 2.14, df = 21, *p* = .04, 95 % CI = .02 to 1.35), engagement (t = 3.82, df = 21, *p* = .02, 95 % CI = .54 to 1.83), and aesthetics (U = 26.0, z = −2.02, *p* = .03). Apps that had a community also scored higher on MARS score (t = 2.29, df = 21, *p* = .03, 95 % CI .07 to 1.35), engagement (t = 2.56, df = 21, *p* < .01, 95 % CI .16 to 1.59), aesthetics (U = 25.5, z = −2.37, *p* = .02), and number of techniques (t = 3.16, df = 21, *p* < .01, 95 % CI 1.54 to 7.49). Apps that used notifications (i.e., prompts and reminders) scored significantly higher in engagement (t = 2.55, df = 21, *p* = .02, 95 % CI .19 to 1.88), and number of techniques (t = 3.07, df = 21, *p* < .01, 95 % CI 1.70 to 8.73). Number of techniques was also higher among apps that required Internet connection to work (t = 2.84, df = 21, *p* < .01, 95 % CI 1.15 to 7.44). Apps that used the most effective techniques combined scored significantly higher on “information quality” (U = 14.00, z = −2.31, *p* = .02). No other differences were identified.

## Discussion

### Principal results

In this paper we described the features of popular weight management apps, analysing their quality and change technique content. App quality was measured through the MARS scale [40], assessing engagement, functionality, aesthetics, and information quality, to provide a more complete evaluation than use of app store user ratings. It is important to note that some apps, although available on both iTunes and GP, offer slightly different features on each operative system. The popular apps examined were overall of moderate quality, but scored higher in terms of functionality and aesthetics. Even though these domains were not significantly associated with user ratings on GP or iTunes, this confirms the idea that people tend to choose well-designed apps that are functional and easy to use [3]. Apps that provided additional interactive features, such as behavioural tracking and semi-automated options, scored significantly higher in engagement, aesthetics and overall app quality, and were also more highly rated on GP. This suggests that reducing the burden associated with manually logging diet or PA may increase engagement, the appeal of an app, and the chances of its repeated use, hence generating a virtuous circle for the app itself.

Aesthetics and functionality should not be the only aspects considered when designing an app [46]. Information quality achieved the lowest score across the MARS domains, indicating an overall lack of evidence-based content, which is consistent with similar reviews [6, 7, 9, 16, 18, 21, 23]. This suggests that developers should invest more in evidence-based, data-driven content, which might improve the overall app quality, regardless of the perceived aesthetic and engagement qualities of the app.

Even though apps vary considerably in the number of techniques included, goal setting, self-monitoring and provision of feedback were the most frequently identified types of change techniques. This is consistent with the literature on weight management interventions generally [43] and similar reviews on apps for PA [8, 13, 14] and PA and diet [11]. From a psychological perspective, then, these apps generally target change mechanisms specified by Control Theory [47]. By contrast, building of behavioural skills was much less evident. For example, “demonstration of behaviour”, which has previously been shown to be associated with positive effects on weight [18], was observed in only 2 of 23 apps. These two apps provided libraries and examples of workouts, commonly found in PA apps [11], but not in general weight-tracking and food logging apps.

This review linked app quality with the presence of types of change techniques and with features of the apps themselves. Using semi-automated tracking (i.e., simplified self-monitoring), having a community, sharing on social media (i.e., offering social support), and using notifications (i.e., behavioural prompts/cues) were features that were associated with higher app quality. This shows that offering features to support specific techniques might improve the perceived functionality, aesthetics and engagement of the app and lead to repeated use. The app quality indicated by MARS scores was positively correlated with number of techniques included, except for information quality, which was only significantly higher only in apps including techniques previously associated with effectiveness. This suggests that perceived information quality is associated with a specific combination of techniques (i.e., goal setting, feedback, and self-monitoring together), which are commonly associated with effectiveness in behavioural interventions. Generally, higher quality apps use a greater range of techniques. This further reinforces the need for developers to incorporate high quality evidence-based content, including specific techniques targeting specified change mechanisms, to improve the overall quality of the app. Future studies should then investigate whether and how the combined use of a variety of techniques found to be effective in other types of weight loss interventions translate into sustained behaviour change supported by an app.

### Strengths and limitations

This review provided a comprehensive evaluation of app quality, described technical features and identified specific techniques in 23 popular weight management apps. For the first time, this review identified positive correlations among app quality dimensions, number of techniques included and app features, which might be useful for users, developers and health care professionals. However, some limitations need to be acknowledged. This review focused explicitly on popular apps, i.e., highly downloaded and rated, and available on both Google Play and iTunes stores to improve comparability among apps currently available on the market. Despite being systematically selected, it remains unclear whether the same features might be found in less popular apps. For feasibility purposes, apps were selected applying thresholds to database filters (e.g., number of downloads, in-app purchases) and a relatively small sample of apps was included. Automatic filtering might have excluded good quality apps that have been misclassified or whose ratings did not reach the thresholds. Apps were evaluated over two days and some features and techniques may have been overlooked as some apps presented new content after repeated use. In addition, considering the dynamic development of apps, app ratings and popularity change over time very quickly. We believe that by focusing on the most popular apps, our conclusions are relatively enduring.

## Conclusions

The popular weight management apps analysed were of moderate quality and provided behavioural tracking features combined with change techniques commonly associated with behaviour change. In addition to functionality and aesthetics, app developers should invest in providing content and employing change techniques known to be effective in changing relevant behaviour patterns in order to improve the user experience and foster behaviour change. Despite growing use of apps in research, additional experimental evaluations of such apps are needed to understand whether the presence of particular content, types of change techniques, is associated with behaviour change.

## Additional files

Additional file 1: Table S1. Number and type of change techniques used and reliability estimates. (PDF 158 kb) Additional file 2: Table S2. MARS coding, reliability estimates and correlations. (PDF 179 kb) Additional file 3: Table S3. Characteristics and features of the reviewed apps. (PDF 121 kb)

## Abbreviations

GP: google play
IQR: interquartile range
MARS: mobile app rating scale
Md: median
PA: physical activity
