# Supplementary material for: A review and content analysis of engagement, functionality, aesthetics, information quality, and change techniques in the most popular commercial apps for weight management
Source: Int J Behav Nutr Phys Act. 2016 Mar 10;13:35. doi: 10.1186/s12966-016-0359-9 (PMC4785735; doi:10.1186/s12966-016-0359-9)
Supplement: Additional file 1: Table S1. — Number and type of change techniques used and reliability estimates. (PDF 158 kb) [file 12966_2016_359_MOESM1_ESM.pdf]

Supplementary Table 1. Number and type of change techniques used and reliability estimates

**Change techniques**	**Calorie Count [1]**	**Calorie Counter [2]**	**CarbsControl [3]**	**Diet Plan [4]**	**Diet Watchers Diary [5]**	**Fast Food Nutri. & Weight Loss [6]**	**FatSecret [7]**	**Foods That Burn Fat [8]**	**Lark [9]**	**MSN Health & Fitness [10]**	**My Diet Coach PRO [11]**	**MY Weight [12]**	**My Diet Diary [13]**	**MyFitnessPal [14]**	**MyNetDiary PRO [15]**	**MyPlate [16]**	**NexTrack [17]**	**Pacer [18]**	**RecStyle [19]**	**Spark People [20]**	**Ultimate Food Value Diary [21]**	**Weilos [22]**	**YouFood [23]**	**Frequency of technique**
Self-monitoring of behaviour [2.3]	1	1	1	1	1	1	1	0	1	1	1	0	1	1	1	1	1	1	0	1	1	1	1	**20**
Self-monitoring of outcomes [2.4]	1	1	1	1	1	0	1	0	0	0	1	1	1	1	1	1	1	1	1	1	1	1	1	**19**
Goal setting (outcome) [1.3]	1	1	1	1	1	0	1	0	1	0	1	1	1	1	1	1	0	1	1	1	1	1	1	**19**
Feedback on outcomes [2.7]	1	1	0	1	1	0	1	0	1	0	1	1	1	1	1	1	1	1	1	1	1	0	0	**17**
Feedback on behaviour [2.2]	1	1	0	1	1	0	1	0	1	1	1	0	1	1	1	1	1	1	0	1	1	0	0	**16**
Review outcome goals [1.7]	1	1	1	0	0	0	1	0	1	0	1	1	1	1	1	1	0	1	1	0	1	1	0	**15**
Goal setting (behaviour) [1.1]	0	1	1	1	0	0	0	1	1	1	1	1	0	1	1	1	0	1	0	0	1	0	0	**13**
Social support (incl. emotional) [3.1] ([a])	1	1	0	0	0	0	1	0	1	0	0	0	1	1	0	1	1	1	0	1	0	0	1	**11**
Review behaviour goals [1.5]	0	1	0	1	0	0	0	0	1	1	1	0	0	1	1	1	0	1	0	0	1	0	0	**10**
Discrepancy behaviour/goal [1.6]	0	1	1	0	0	0	1	0	0	1	1	0	0	0	1	1	0	1	0	0	1	0	0	**9**
Instruction on behaviour [4.1]	0	1	0	1	0	0	1	1	0	1	0	0	0	0	1	1	0	0	0	1	0	0	1	**9**
Social comparison [6.2]	1	0	0	0	0	0	1	0	0	0	0	0	0	1	0	0	1	1	0	1	0	1	1	**8**
Social reward [10.4]	1	0	0	0	0	0	0	0	1	0	0	0	0	1	0	0	1	0	0	1	0	1	1	**7**
Information on health consequences [5.1]	0	1	0	1	0	0	0	0	0	1	0	0	1	0	1	0	1	0	0	1	0	0	0	**7**
Prompts/cues [7.1] ([b])	0	0	0	1	0	0	0	0	1	0	1	0	1	0	1	1	0	0	0	0	0	0	0	**6**
Information about antecedents [4.2] ([c])	0	0	0	1	0	0	0	1	0	1	1	0	0	0	1	0	0	0	0	0	0	0	0	**5**
Behaviour substitution [8.2] ([d])	0	1	0	1	0	0	0	1	0	0	1	0	0	0	0	0	0	0	0	1	0	0	0	**5**
Non specific reward [10.3]	0	0	0	0	0	0	0	0	0	0	1	0	0	0	0	0	1	0	0	1	0	1	0	**4**
Reference to credible sources [9.1] ([e])	0	1	0	0	0	0	0	0	1	1	0	0	0	0	1	0	0	0	0	0	0	0	0	**4**
Habit formation [8.3] ([f])	0	0	0	0	0	0	0	0	0	0	1	0	0	0	0	0	0	1	0	0	1	0	0	**3**
Commitment [1.9]	0	1	0	0	0	0	0	0	0	0	1	0	0	0	0	0	0	0	0	0	0	0	0	**2**
Demonstration of behaviour [6.1] ([g])	0	0	0	0	0	0	0	0	0	1	0	0	0	0	0	1	0	0	0	0	0	0	0	**2**

**Change techniques**	**Calorie Count [1]**	**Calorie Counter [2]**	**CarbsControl [3]**	**Diet Plan [4]**	**Diet Watchers Diary [5]**	**Fast Food Nutri. & Weight Loss [6]**	**FatSecret [7]**	**Foods That Burn Fat [8]**	**Lark [9]**	**MSN Health & Fitness [10]**	**My Diet Coach PRO [11]**	**MY Weight [12]**	**My Diet Diary [13]**	**MyFitnessPal [14]**	**MyNetDiary PRO [15]**	**MyPlate [16]**	**NexTrack [17]**	**Pacer [18]**	**RecStyle [19]**	**Spark People [20]**	**Ultimate Food Value Diary [21]**	**Weilos [22]**	**YouFood [23]**	**Frequency of technique**
Graded tasks [8.7] (h)	0	0	0	1	0	0	0	0	0	0	1	0	0	0	0	0	0	0	0	0	0	0	0	**2**
Action planning [1.4] (i)	0	0	0	0	0	0	0	0	0	0	1	0	0	0	0	0	0	0	0	0	0	0	0	**1**
**Total number of techniques**	**9**	**15**	**6**	**13**	**5**	**1**	**10**	**4**	**11**	**10**	**17**	**5**	**9**	**11**	**14**	**13**	**9**	**12**	**4**	**12**	**10**	**7**	**7**	
**Reliability estimates**																								
Krippendorff's alpha	.78	.62	.64	.83	1.00	1.00	.84	.85	.28	1.00	.60	.88	.94	.70	.69	.54	.86	.89	.85	.75	.88	.77	0.94	0.79
Agreement	92	84	94	92	100	100	94	98	70	100	80	98	98	90	88	84	94	96	98	90	96	94	98	93

Notes: The change techniques are sorted by frequency of occurrence. Change techniques found to be associated with effective behaviour change leading to weight loss or weight maintenance in previous research are highlighted in ***italics and bold***. Some apps, such as *Lark*, *My Diet Coach PRO*, *NexTrack*, *MyNetDiary* and *Calorie Counter*, may contain a higher number of techniques than estimated as they include healthy tips and strategies that are only shown after repeated and longer use.

(a) The social support category includes any type of support such as "unspecified" and "emotional" social support as these distinctions could not be drawn in coding app content. Practical social support was not found in these apps.

(b) Prompts and cues: This technique was attributed to apps that presented notifications and reminders when they aimed to cue the behaviour, following the BCT taxonomy (v1) definition. Reminders and notifications that prompted for logging in the app were not coded.

(c) Instruction on antecedents: Coded when apps presented tips and hints that identified social or environmental situations or events that could influence the performance of the behaviour, such as keeping a log for moments of snacking.

(d) Behaviour substitution: Coded when apps presented hints and strategies to avoid the unwanted behaviour (e.g., overeating, snacking).

(e) Reference to credible sources: Coded when apps referenced evidence to support presented advice or information, for example guidelines or studies that supported the apps content.

(f) Habit formation: It is possible to view all apps that employ tracking, food logging, or weight-tracking app as incorporating practice and so building new habits. However, this category was only coded only when the app prompted explicit rehearsal and repetition of the behaviour.

(g) Demonstration of behaviour: Included because associated with positive effects on weight in behavioural interventions, this technique was coded in apps that offered a library of videos with workouts that demonstrated how to perform various physical activities.

(h) Graded tasks: As with "habit formation", this technique can be considered an implicit element of any app. Coded only when the app explicitly presented progressively more difficult tasks leading towards a behavioural goal (e.g., food logging and weight tracking).

(i) Action planning: Coded only when the app explicitly prompted a detailed planning of the behaviour, including context, frequency and time (duration and intensity), following the BCT taxonomy's (v1) definition. This specific definition led to only two instances being observed.
