# Supplementary material for: A review and content analysis of engagement, functionality, aesthetics, information quality, and change techniques in the most popular commercial apps for weight management
Source: Int J Behav Nutr Phys Act. 2016 Mar 10;13:35. doi: 10.1186/s12966-016-0359-9 (PMC4785735; doi:10.1186/s12966-016-0359-9)
Supplement: Additional file 2: Table S2. — MARS coding, reliability estimates and correlations. (PDF 179 kb) [file 12966_2016_359_MOESM2_ESM.pdf]

Supplementary Table 2. MARS coding, reliability estimates and correlations

App	Coders	1. Entertainment	2. Interest	3. Customisation	4. Interactivity	5. Target group	6. Performance	7. Ease of use	8. Navigation	9. Gestural design	10. Layout	11.Graphics	12. Visual appeal	13. Accuracy of app description	14. Goals	15. Quality of information	16. Quantity of information	17. Visual information	18. Credibility	19. Evidence base	Engagement score	Functionality score	Aesthetics score	Information quality score	Total MARS score	*Kalpha*
Calorie Count [1]	MB	1	2	4	3	3	1	1	1	4	2	1	1	3	4	1	1	3	1	1	2.6	1.8	1.3	2.0	**1.9**	***.79***
	SVB	2	2	5	3	3	1	2	2	5	3	2	2	2	4	1	1	4	1	1	3.0	2.5	2.3	2.0	**2.5**	
Calorie Counter [2]	SVB	1	1	2	3	3	3	4	3	5	3	3	3	4	3	0	0	5	1	0	2.0	3.8	3.0	1.9	**2.7**	***.82***
	MB	2	2	3	3	3	4	3	3	4	4	4	4	5	3	0	0	4	1	0	2.6	3.5	4.0	1.9	**3.0**	
CarbsControl [3]	MB	1	1	3	1	4	3	3	3	3	1	1	1	4	3	0	0	1	1	0	2.0	3.0	1.0	1.3	**1.8**	***.87***
	SVB	1	1	3	2	4	4	4	4	3	3	2	1	5	3	0	0	0	1	0	2.2	3.8	2.0	1.3	**2.3**	
Diet Plan [4]	MB	1	1	3	2	3	2	1	1	3	1	2	1	4	2	3	4	2	2	1	2.0	1.8	1.3	2.6	**1.9**	***.70***
	SVB	1	2	3	3	3	1	1	2	3	1	2	2	3	1	2	4	2	1	1	2.4	1.8	1.7	2.0	**2.0**	
Diet Watchers Diary [5]	MB	1	2	3	3	3	1	1	2	3	1	2	2	3	1	2	4	2	1	1	2.4	1.8	1.7	2.0	**2.0**	***.91***
	SVB	1	1	3	2	3	2	1	1	3	1	2	1	4	2	3	4	2	2	1	2.0	1.8	1.3	2.6	**1.9**	
Fast Food Nutrition & Weight Loss [6]	MB	1	1	1	4	3	4	1	3	5	3	2	3	3	0	4	3	5	1	0	2.0	3.3	2.7	2.3	**2.6**	***.92***
	SVB	1	1	1	5	3	3	1	3	5	4	3	3	4	0	5	2	5	1	0	2.2	3.0	3.3	2.4	**2.7**	
Fat Burning Foods [7]	MB	1	2	1	1	2	2	4	4	5	2	1	1	4	0	3	2	0	1	0	1.4	3.8	1.3	1.4	**2.0**	***.88***
	SVB	1	1	1	1	2	2	5	5	5	2	2	2	5	0	3	1	0	1	0	1.2	4.3	2.0	1.4	**2.2**	
FatSecret [8]	MB	1	3	3	3	3	5	4	4	4	4	3	4	4	4	3	3	4	1	1	2.6	4.3	3.7	2.9	**3.3**	***.72***
	SVB	1	4	4	3	3	5	5	5	5	5	3	3	5	4	3	2	3	1	1	3.0	5.0	3.7	2.7	**3.6**	
Lark [9]	MB	5	5	4	5	3	5	4	4	5	5	4	5	5	0	3	3	4	5	0	4.4	4.5	4.7	2.9	**4.1**	***.73***
	SVB	5	5	5	5	3	5	5	5	5	5	3	4	5	0	4	3	3	4	0	4.6	5.0	4.0	2.7	**4.1**	
MSN Health & Fitness [10]	MB	3	4	3	3	3	4	4	4	4	4	5	4	2	3	5	4	4	2	0	3.2	4.0	4.3	2.9	**3.6**	***.75***

App	Coders	1. Entertainment	2. Interest	3. Customisation	4. Interactivity	5. Target group	6. Performance	7. Ease of use	8. Navigation	9. Gestural design	10. Layout	11.Graphics	12. Visual appeal	13. Accuracy of app description	14. Goals	15. Quality of information	16. Quantity of information	17. Visual information	18. Credibility	19. Evidence base	Engagement score	Functionality score	Aesthetics score	Information quality score	Total MARS score	*Kalpha*
	SVB	4	4	2	2	3	4	4	5	5	5	5	5	3	3	5	5	5	3	0	3.0	4.5	5.0	3.4	**4.0**	
My Diet Coach [11]	MB	5	5	5	5	5	5	5	5	5	4	5	5	5	5	5	4	5	1	0	5.0	5.0	4.7	3.6	**4.6**	***.87***
	SVB	5	5	5	5	5	5	5	5	5	5	5	5	5	5	5	4	5	1	0	5.0	5.0	5.0	3.6	**4.6**	
My Diet Diary [12]	MB	1	4	4	4	3	4	4	3	3	4	5	4	5	2	0	0	4	1	0	3.2	3.5	4.3	1.7	**3.2**	***.73***
	SVB	2	2	4	3	3	4	5	4	5	5	5	4	5	2	0	0	4	3	0	2.8	4.5	4.7	2.0	**3.5**	
MY Weight [13]	MB	1	2	4	2	3	5	3	3	4	3	2	2	5	3	0	0	5	1	0	2.4	3.8	2.3	2.0	**2.6**	***.83***
	SVB	1	1	3	3	3	2	4	4	5	3	2	2	5	3	0	0	4	1	0	2.2	3.8	2.3	1.9	**2.5**	
MyFitnessPal [14]	MB	2	3	4	5	3	4	5	5	5	4	5	5	4	4	3	3	2	1	1	3.4	4.8	4.7	2.6	**3.8**	***.70***
	SVB	1	4	5	5	3	5	4	3	5	5	5	5	5	5	4	2	1	1	1	3.6	4.3	5.0	2.7	**3.9**	
MyNetDiary - Pro [15]	MB	2	4	5	4	3	3	5	5	5	5	5	5	4	4	0	0	5	2	0	3.6	4.5	5.0	2.1	**3.8**	***.82***
	SVB	3	3	4	3	3	4	4	5	5	5	4	4	3	4	0	0	5	1	0	3.2	4.5	4.3	1.9	**3.5**	
MyPlate [16]	MB	2	4	4	4	3	5	5	5	5	5	5	5	5	4	0	0	5	1	0	3.4	5.0	5.0	2.1	**3.9**	***.67***
	SVB	2	3	3	3	3	2	3	5	5	4	4	3	5	5	0	0	5	1	0	2.8	3.8	3.7	2.3	**3.1**	
NexTrack [17]	MB	5	4	5	5	3	3	4	4	5	5	4	3	4	0	0	0	4	1	0	4.4	4.0	4.0	1.3	**3.4**	***.86***
	SVB	5	5	4	5	3	2	3	3	5	4	4	4	3	0	0	0	4	1	0	4.4	3.3	4.0	1.1	**3.2**	
Pacer [18]	MB	1	3	4	3	3	3	5	5	5	5	5	4	5	3	0	2	5	2	0	2.8	4.5	4.7	2.4	**3.6**	***.82***
	SVB	2	2	3	2	3	3	4	5	5	4	4	3	5	5	0	1	5	1	0	2.4	4.3	3.7	2.4	**3.2**	
RecStyle [19]	MB	1	1	2	3	3	4	3	3	5	3	3	3	5	3	0	0	3	1	0	2.0	3.8	3.0	1.7	**2.6**	***.84***
	SVB	2	2	3	3	3	3	4	4	5	4	2	3	5	3	0	0	4	1	0	2.6	4.0	3.0	1.9	**2.9**	
SparkPeople [20]	MB	4	5	5	4	3	5	4	5	5	4	5	4	5	4	5	5	5	3	1	4.2	4.8	4.3	4.0	**4.3**	***.79***
	SVB	4	5	5	3	3	5	3	5	5	5	5	5	5	5	5	5	5	3	1	4.0	4.5	5.0	4.1	**4.4**	

App	Coders	1. Entertainment	2. Interest	3. Customisation	4. Interactivity	5. Target group	6. Performance	7. Ease of use	8. Navigation	9. Gestural design	10. Layout	11.Graphics	12. Visual appeal	13. Accuracy of app description	14. Goals	15. Quality of information	16. Quantity of information	17. Visual information	18. Credibility	19. Evidence base	Engagement score	Functionality score	Aesthetics score	Information quality score	Total MARS score	*Kalpha*
Ultimate Food Value Diary [21]	SVB	1	1	5	2	3	1	3	3	3	1	2	1	4	3	0	0	3	1	0	2.4	2.5	1.3	1.6	**2.0**	***.65***
	MB	2	4	5	2	3	4	2	3	4	2	3	2	5	3	0	0	2	1	0	3.2	3.3	2.3	1.6	**2.6**	
Weilos [22]	MB	4	3	4	5	3	4	3	4	4	3	5	4	4	3	0	0	1	1	0	3.8	3.8	4.0	1.3	**3.2**	***.70***
	SVB	3	3	3	4	3	3	4	5	5	4	4	3	5	4	0	0	0	1	0	3.2	4.3	3.7	1.4	**3.1**	
YouFood [23]	MB	3	4	3	3	3	2	3	4	3	4	5	4	4	0	0	0	4	1	0	3.2	3.0	4.3	1.3	**3.0**	***.80***
	SVB	4	4	4	3	3	3	4	4	4	4	4	4	5	0	0	0	5	1	0	3.6	3.8	4.0	1.6	**3.2**	
Correlations of scores among coders (p < .01)																					***.86***	***.72***	***.81***	***.93***	***.87***	

Notes: Kalpha = Krippendorff's alpha.
