# Supplementary material for: A review and content analysis of engagement, functionality, aesthetics, information quality, and change techniques in the most popular commercial apps for weight management
Source: Int J Behav Nutr Phys Act. 2016 Mar 10;13:35. doi: 10.1186/s12966-016-0359-9 (PMC4785735; doi:10.1186/s12966-016-0359-9)
Supplement: Additional file 3: Table S3. — Characteristics and features of the reviewed apps. (PDF 121 kb) [file 12966_2016_359_MOESM3_ESM.pdf]

Supplementary Table 3. Characteristics and features of the reviewed apps

App [ID]	Source	Price (USD) (GP/iTunes)	Version tested (GP/iTunes)	Average user rating (GP/iTunes)	Total user ratings (GP/iTunes)	Behaviour focus and Tracking	In-app behavioural tracking details	Number of change techniques	MARS score	Allows sharing	Has community	Requires login	Privacy	Works in background	Has notifications	Needs web access	Total features
CalorieCount [1]	GP/iT	0 / 0	4.2.5 / 3.18	4 / 4.5	6951 / 18354	WDPA	Mixed (manual weight tracking; simplified dietary tracking (barcode scanner); manual PA tracking)	9	2.2	1	1	1	0	1	1	0	5
CalorieCounter [2]	iT	0 / 0	2.0 / 4.2.0	3.9 / 4.0	906 / 4323	WDPA	Manual only (weight, dietary, and PA tracking)	15	2.8	0	1	1	1	0	1	1	5
CarbsControl [3]	iT	2.99 / 2.99	4.7 / 7.1	3.4 / 4.0	124 / 1239	WD	Manual only (weight and dietary tracking)	6	2.1	0	0	0	0	1	0	0	1
Diet Plan [4]	GP	0 / 0	1.1.1 / 1.3	4.1 / N/A	6063 / N/A	WDPA	Manual only (weight, dietary, and PA tracking)	13	1.9	0	0	1	1	1	1	1	5
Diet Watchers Diary [5]	GP	2.39 / 2.99	5.5.0 / 5.2.1	4.6 / 3.5	1409 / 15	WDPA	Manual only (weight, dietary, and PA tracking)	5	1.9	0	0	0	0	1	1	0	2
Fast Food Nutrition & Weight Loss [6]	GP/iT	2.99 / 2.99	39.0 / 1.49	4.5 / 4.5	750 / 925	Not tracking: Food info	None	1	2.6	0	0	0	0	0	0	0	0

App [ID]	Source	Price (USD) (GP/iTunes)	Version tested (GP/iTunes)	Average user rating (GP/iTunes)	Total user ratings (GP/iTunes)	Behaviour focus and Tracking	In-app behavioural tracking details	Number of change techniques	MARS score	Allows sharing	Has community	Requires login	Privacy	Works in background	Has notifications	Needs web access	Total features
FatSecret [7]	GP	0 / 0	4.1.2.7 / 6.1.1	4.2 / 3.5	152547 / 2498	WDPA	Manual only (weight, dietary, and PA tracking)	10	3.5	0	1	1	1	0	1	1	5
Foods That Burn Fat [8]	GP	0 / 0	1.0 / 3.0	4.0 / 0.0	1125 / 0	Not tracking: Food info	None	4	2.1	0	0	0	0	0	0	1	1
Lark [9]	iT	0 / 0	2.4.1 / 2.2.0	4.1 / 4.5	917 / 890	WDPA	Mixed (manual and simplified weight tracking (e.g., Withings scale integration); manual dietary tracking; manual and automatic PA tracking through (phone motion sensor)	11	4.1	0	0	1	0	1	1	0	3

App [ID]	Source	Price (USD) (GP/iTunes)	Version tested (GP/iTunes)	Average user rating (GP/iTunes)	Total user ratings (GP/iTunes)	Behaviour focus and Tracking	In-app behavioural tracking details	Number of change techniques	MARS score	Allows sharing	Has community	Requires login	Privacy	Works in background	Has notifications	Needs web access	Total features
MSN Health & Fitness [10]	GP	0 / 0	1.2.0 / 1.2.64	4.4 / 4.5	1348 / 204	DPA	Mixed (manual dietary tracking; manual and simplified PA tracking (phone motion sensor)	10	3.8	0	0	0	0	0	0	1	1
My Diet Coach PRO [11]	GP/iT	3.99 / 3.99	4.2.8 / 7.3.1	4.6 / 4.5	7588 / 1634	WDPA	Manual only (weight, dietary, and PA tracking)	17	4.6	1	1	0	0	1	1	1	5
MY Weight [12]	iT	0 / 0	3.7.7 / 3.7.4	4.3 / 4.5	25958 / 641	W	Manual weight tracking	5	2.6	0	0	0	1	1	0	0	2
MyDietDiary [13]	GP/iT	0 / 0	1.10.4 / 5.5.0	4.2 / 4.0	15256 / 753	WDPA	Mixed (Manual and simplified weight tracking through digital scales; PA tracking via phone motion sensor)	9	3.3	0	1	1	1	0	1	1	5

App [ID]	Source	Price (USD) (GP/iTunes)	Version tested (GP/iTunes)	Average user rating (GP/iTunes)	Total user ratings (GP/iTunes)	Behaviour focus and Tracking	In-app behavioural tracking details	Number of change techniques	MARS score	Allows sharing	Has community	Requires login	Privacy	Works in background	Has notifications	Needs web access	Total features
MyFitnessPal [14]	GP/iT	0 / 0	5.0 / 6.6.1	4.6 / 4.5	836597 / 438279	WDPA	Mixed (Manual and simplified weight tracking through digital scales, manual and simplified dietary tracking via barcode scanner, PA tracking via phone motion sensor)	11	3.9	1	1	1	1	1	1	0	6
MyNetDiary PRO [15]	iT	0 / 3.99	3.1.0 / 4.65	4.4 / 4.5	6493 / 13330	WDPA	Mixed (Manual and simplified dietary via barcode scanner, and activity tracking via phone motion sensor)	14	3.6	1	1	0	1	1	1	1	6
MyPlate [16]	iT	0 / 0	2.0.0(0) / 5.4	2.9 / 4.5	1352 / 10301	WDPA	Manual only (weight, dietary, and PA tracking)	13	3.5	0	1	1	0	1	1	1	5

App [ID]	Source	Price (USD) (GP/iTunes)	Version tested (GP/iTunes)	Average user rating (GP/iTunes)	Total user ratings (GP/iTunes)	Behaviour focus and Tracking	In-app behavioural tracking details	Number of change techniques	MARS score	Allows sharing	Has community	Requires login	Privacy	Works in background	Has notifications	Needs web access	Total features
NexTrack [17]	iT	0 / 0	2.7.85 / 2.5.4	4.4 / 4.5	20214 / 14091	WPA	Mixed (manual weight tracking; simplified PA tracking via motion sensor)	9	3.3	1	1	1	1	1	1	1	7
Pacer [18]	GP/iT	0 / 0	p2.7.2 / 3.5.2	4.1 / 4.5	10163 / 1650	WPA	Mixed (manual weight tracking; manual and simplified dietary tracking through integration with MyFitnessPal); manual and simplified PA tracking via motion sensor)	12	3.4	0	1	1	0	1	1	1	5
RecStyle [19]	GP	0 / 0	3.0.7 / 2.0.3	4.2 / 4.5	7096 / 11	W	Manual weight tracking	4	2.7	0	0	0	1	1	1	0	3

App [ID]	Source	Price (USD) (GP/iTunes)	Version tested (GP/iTunes)	Average user rating (GP/iTunes)	Total user ratings (GP/iTunes)	Behaviour focus and Tracking	In-app behavioural tracking details	Number of change techniques	MARS score	Allows sharing	Has community	Requires login	Privacy	Works in background	Has notifications	Needs web access	Total features
SparkPeople [20]	GP/iT	0 / 0	4.04 / 4.01	4.2 / 4.0	3784 / 603	WDPA	Mixed (manual and simplified dietary via barcode scanner, and manual activity tracking)	12	4.4	1	1	1	0	1	1	1	6
Ultimate Food Value Diary [21]	iT	3.99 / 3.99	5.5.0 / 3.1.3	4.7 / 4.5	1770 / 2113	WDPA	Manual only (weight, dietary, and PA tracking)	10	2.3	0	1	0	1	0	1	1	4
Weilos [22]	iT	0 / 0	1.0.0 / 2.4.4	2.4 / 5.0	31 / 1684	Not tracking: Weight loss community	None	7	3.2	1	1	1	0	0	1	1	5
YouFood [23]	GP/iT	0 / 0	2.6.5 / 2.6.2	4.5 / 5	1468 / 811	Not tracking: Weight loss community	None	7	3.1	1	1	1	0	0	1	1	5

Notes: GP, Google Play; iT, iTunes; MARS, Mobile App Rating Scale; PA, physical activity; Behavioural focus: WDPA, Weight, diet and PA; WPA, Weight and PA; WD, Weight and diet; W, Weight only.
